# The Role of HPV Genotyping, Cytology, and Methylation in the Triage of High-Risk HPV-Positive Patients

**DOI:** 10.3390/biomedicines13051139

**Published:** 2025-05-08

**Authors:** Anastasia Mortaki, Athanasios Douligeris, Maria-Anastasia Daskalaki, Eleni-Sivylla Bikouvaraki, Eirini Louizou, George Daskalakis, Alexandros Rodolakis, Themos Grigoriadis, Kalliopi I Pappa

**Affiliations:** 1 1st Department of Obstetrics and Gynecology, National and Kapodistrian University of Athens, “Alexandra” General Hospital, 11528 Athens, Greece; anastasiamort@gmail.com (A.M.); anastasia.daskalaki00@gmail.com (M.-A.D.); gdaskalakis@yahoo.com (G.D.); a.rodolaki@gmail.com (A.R.); tgregos@yahoo.com (T.G.); kpappa@med.uoa.gr (K.I.P.); 2 Laboratory of Cell and Gene Therapy, Centre of Basic Research, Biomedical Research Foundation of the Academy of Athens (BRFAA), 11527 Athens, Greece; bsivylla@yahoo.com; 3 Department of Cytogenetics and Molecular Genetics, Bioiatriki, Group of Health Sciences, 11528 Athens, Greece; louizoueirini1978@gmail.com

**Keywords:** human papillomavirus, high-grade squamous intraepithelial lesions, DNA methylation, FAM19A4, hsa-miR124-2, cervical cytology, cervical cancer screening

## Abstract

**Background/Objectives**: This study evaluates the diagnostic performance of DNA methylation testing, alone and in combination with cervical cytology, for high-grade squamous intraepithelial lesion (HSIL) detection. **Methods**: A prospective study was conducted on 170 high-risk HPV (hr-HPV)-positive women. DNA methylation (QIAsure^®^) and cervical cytology were performed prior to cervical large loop excision of the transformation zone (LLETZ). Sensitivity, specificity, and area under the curve (AUC) metrics were calculated, including stratified analyses for HPV16/18 and other hr-HPV genotypes. **Results**: DNA methylation alone achieved a sensitivity of 69.7%, specificity of 79%, and an AUC of 0.796 for HSIL detection. The combination of cervical cytology and DNA methylation improved sensitivity to 94.7%, specificity to 76.3%, and AUC to 0.860. Stratification by HPV genotype revealed that in HPV16/18-positive cases, DNA methylation alone reached an AUC of 0.790, while the combination with cytology significantly enhanced performance to 0.917. DNA methylation alone demonstrated an AUC of 0.744 for other hr-HPV types, and the combined approach achieved an AUC of 0.849. Specificity for the combined approach was notably higher in HPV16/18-positive women (88.9%) than in other hr-HPV cases (72.4%), whereas the sensitivity of the combined approach was significantly higher in both groups (94.5% vs. 95%, respectively). **Conclusions**: The integration of DNA methylation with cervical cytology significantly enhances diagnostic accuracy for CIN2+ lesions, especially in HPV16/18-positive cases. However, the comparatively lower AUC and specificity observed in other hrHPV types suggest the need for further optimization to enhance accuracy in non-16/18 infections. These findings support the integration of methylation-based testing with cytology as a valuable triage strategy for improving cervical cancer screening and patient management.

## 1. Introduction

Human Papillomavirus (HPV) is the primary cause of squamous cell cervical cancer, with nearly 99.7% of cases related to high-risk HPV (hr-HPV) types [1]. Although HPV infection is necessary for the transformation of cervical epithelial cells, it alone is insufficient to cause cervical cancer. The development of carcinogenesis typically involves a combination of HPV infection and other molecular events [2,3]. Effective prevention strategies include primary prevention through vaccination against nine high-risk genotypes, covering 90% of cervical cancer cases [4,5]. On the other hand, secondary prevention through identification and treatment of precancerous lesions and tertiary prevention focusing on reducing the disease impact through appropriate treatments or palliative care [6,7].

Cervical cancer screening initially relied on the Papanicolaou (Pap) test and later incorporated the HPV test to improve sensitivity. However, HPV testing has an age-dependent low specificity compared to the Pap test [8]. Current screening guidelines recommend the use of the HPV test as the primary tool, either alone or in combination with the Pap test, followed by further triage. Women testing positive for HPV16 and/or HPV18 are directly referred for colposcopy, while those positive for other hr-HPV types undergo cytological triage [9]. Nevertheless, given the limitations of cytology’s sensitivity and the low specificity of the HPV testing for detecting high-grade CIN lesions, current research focuses on developing combined strategies with novel tests to improve sensitivity and specificity on triaging patients with positive hr-HPV types [10].

Among emerging strategies, host cell DNA methylation testing has shown promising results. DNA methylation is an epigenetic mechanism involving the addition of methyl groups to cytosine residues within CpG islands, leading to gene silencing. Persistent hr-HPV infection can induce aberrant methylation of tumor suppressor genes and other regulatory genes, promoting cellular transformation, immune evasion, and progression to cervical intraepithelial neoplasia and cancer [11]. The accumulation of methylation changes reflects the severity and duration of HPV-induced cellular alterations, thus serving as a biological marker of disease progression.

Several methylation markers have been studied for triage applications in hr-HPV-positive women. Markers such as FAM19A4 and hsa-miR124-2 have been extensively validated and incorporated into commercially available tests (e.g., QIAsure^®^), demonstrating consistent performance in detecting CIN2+ lesions across diverse populations. Their main advantages include high reproducibility, objective molecular readout, and applicability even in samples with low cellularity, such as self-collected specimens. However, their performance can vary depending on patient age and HPV genotype, highlighting the need for optimization in certain subgroups [12]. Additional methylation markers, including PAX1, JAM3, CADM1, and MAL, have also been evaluated for their potential triage utility. Notably, recent studies by Fei et al. and Li et al. have demonstrated that PAX1 and JAM3 methylation are strongly associated with persistent HPV infection and can effectively predict the risk of cervical high-grade lesions, particularly among HPV16/18-positive women [13,14].

The present study aims to evaluate the DNA methylation of FAM19A4 and hsa-miR124-2 host genes as a triage test, alone or in combination with the Pap test, in hr-HPV-positive women.

## 2. Materials and Methods

### 2.1. Study Design & Patient Selection

This was a prospective, single-center, observational, cohort study conducted under conditions of routine clinical practice between March 2019 and December 2020. This study was designed according to the Declaration of Helsinki and its relevant amendments. Institutional Ethics Committee/Review Board approval was obtained prior to the initiation of the study (Number 238/18-03-2019) on 18 March 2019. All patients were informed about the study’s objectives and the procedures and signed an informed consent. Patients between 21 and 70 years old, with a recent (within 2 months) abnormal cytology, who were scheduled for cervical conization, were included in the study. Patients under immunosuppressive treatment, pregnant, with a medical history of cervical cancer or pelvic radiation therapy, or insufficient material for test diagnosis were excluded.

For the purposes of this study, all patients were referred to our Colposcopy Clinic following an initial liquid-based cervical cytology, defined as Pap test 1, with abnormal results of at least atypical squamous cells of undetermined significance (>ASCUS).

**Figure 1:**
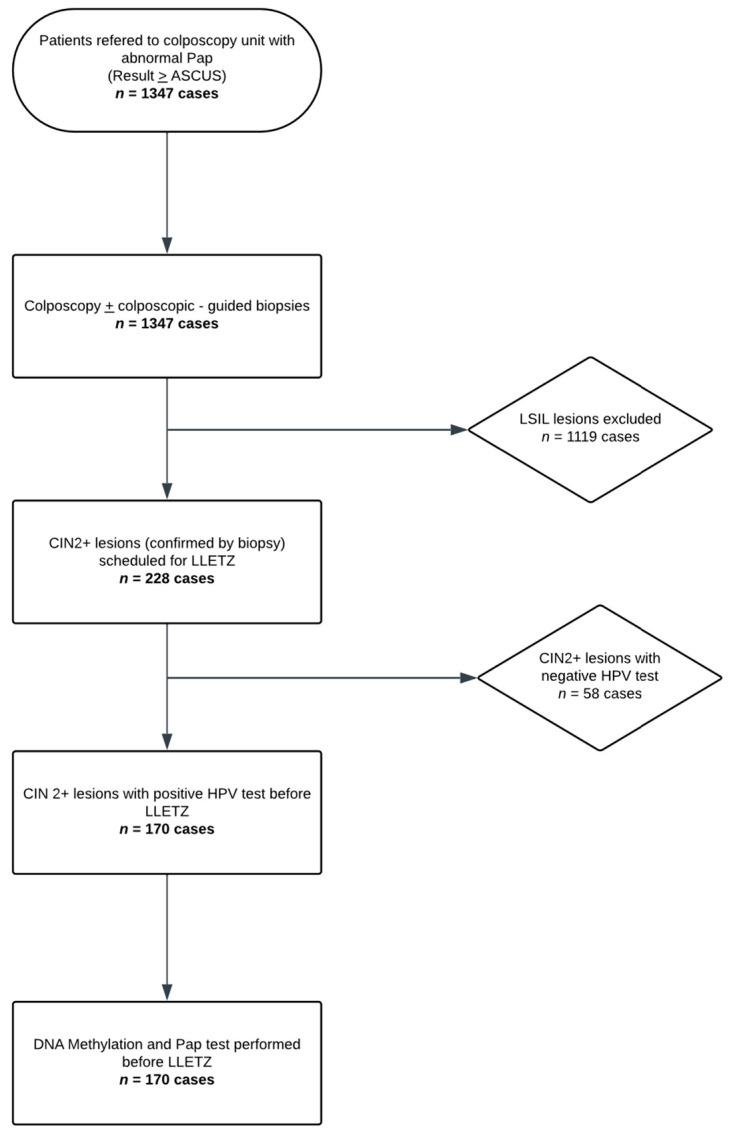
Study Flow Diagram (Flowchart illustrating the study population and sample processing).

Colposcopy was performed by an experienced colposcopist, and colposcopy-guided biopsies were obtained in each subject, with a minimum of one and a maximum of four biopsies per patient. All women requiring large loop excision of the transformation zone (LLETZ) based on histological findings were included in the study. On the day of the procedure, exactly prior to the LLETZ, a liquid-based cervical cytology sample (ThinPrep^®^, Hologic Inc., Marlborough, MA, USA)) was collected. A repeat cervical cytology (Pap test 2), the HPV test (Roche COBAS 4800 HPV system, Roche Molecular Systems, Inc., Pleasanton, CA, USA), and a methylation test (QIAsure^®^, QIAGEN GmbH, Hilden, Germany), when the HPV test was positive, were performed on the ThinPrep^®^ sample. The aforementioned process is schematically represented in the flowchart of Figure 1.

### 2.2. Definitions

The initial cervical cytology (Pap test 1) was performed before colposcopy and the colposcopy-guided biopsies, as well as the second cervical cytology (Pap test 2) was performed before LLETZ. Cytology results were categorized as ASCUS, low-grade squamous intraepithelial lesion (LSIL), atypical squamous cells cannot exclude HSIL (ASC-H), high-grade squamous intraepithelial lesion (HSIL), atypical glandular cells (AGC), and suspected infiltration.

Colposcopic evaluations were classified into LSIL, LSIL with unsatisfactory colposcopy (LSIL UN), HSIL, and HSIL with unsatisfactory colposcopy (HSIL UN). Pathology reports from colposcopy-guided biopsies and LLETZ were recorded as CIN 1, CIN 2, CIN 3, or carcinoma (Ca). HPV test results identified positive cases for HPV 16, HPV 18, or other high-risk genotypes. DNA methylation tests were considered positive if either or both FAM19A4 and hsa-miR124-2 were hypermethylated, and negative if both genes were not. The worst histological diagnosis from colposcopy-guided biopsies or LLETZ biopsies was considered the overall histological diagnosis for each case.

In our study, sensitivity (Se), specificity (Sp), positive predictive value (PPV), and negative predictive value (NPV) are defined and calculated differently from their conventional definitions. Typically, these metrics are determined by comparing a test’s ability to distinguish between healthy individuals and those with a disease. However, in our study, we use populations with low-grade and high-grade lesions to determine these values.

- Sensitivity (Se): The proportion of high-grade lesions correctly identified by the test:

(1) $$Se=\;\frac{True\;Positives\;(HSIL-Ca)}{True\;positives\;(HSIL-Ca)}+False\;Negatives\;(HSIL-Ca)$$

- Specificity (Sp): The proportion of low-grade lesions correctly identified by the test:

(2) $$Sp=\frac{\;True\;Negatives\;(LSIL)}{\;True\;Negatives\;(LSIL)}+False\;Positives\;(LSIL)$$

- Positive Predictive Value (PPV): The probability that lesions identified as high-grade by the test are truly high-grade:

(3) $$PPV=\frac{True\;Positives\;(HSIL-Ca)}{True\;Positives\;(HSIL-Ca)}+False\;Positives\;(LSIL)$$

- Negative Predictive Value (NPV): The probability that lesions identified as low-grade by the test are truly low-grade:

(4) $$NPV=\frac{True\;Negatives\;(LSIL)}{True\;Negatives\;(LSIL)}+False\;Negatives\;(HSIL-Ca)$$

### 2.3. Predetermined Outcomes

Differences in the sensitivity and specificity for detecting HSIL and/or cancer in patients with positive hr-HPV DNA tests using methylation assays compared to cytology were considered as the primary outcomes of the present study. Differences in these metrics between the combination of methylation and cytology versus cytology alone were also analyzed. As secondary outcomes, the same comparisons were considered within the subgroups of women with HPV DNA positive for types 16/18 and those with other hr-HPV subtypes.

### 2.4. Statistical Analysis

Data were expressed as mean ± SD for continuous variables, and as frequencies and percentages for categorical data. The Kolmogorov–Smirnov test was utilized for the normality analysis of the parameters. The diagnostic ability of the Pap test, colposcopy, and DNA methylation test for the detection of CIN2 or worse (CIN2+) was evaluated using sensitivity, specificity, positive predictive value (PPV), negative predictive value (NPV), and accuracy. Comparison between them was performed using the Binary Diagnostic Paired Samples test as proposed by Nam et al. [15] with the NCSS Statistical Software 2021 vr 21.0.3. ROC curve analysis was conducted to calculate the area under the curve (AUC) with 95% confidence intervals, and comparisons between the methods were performed to assess statistical significance. All statistical tests were two-sided, and statistical significance was set at *p* < 0.05. All analyses were carried out using IBM SPSS Statistics for Windows, Version 21.0. Armonk, NY, USA.

A total sample size of 140 achieved 86% power to detect a change in sensitivity from 0.7 to 0.9 using a one-sided binomial test and 100% power to detect a change in specificity from 0.5 to 0.7 using a one-sided binomial test. The target significance level was 0.05. The prevalence of the disease was 0.2. Sample size estimation was performed using the PASS 11.0.8 program (NCSS, LLC, Kaysville, UT, USA).

## 3. Results

From a total cohort of 1347 women, we stratified 228 patients who were scheduled for LLETZ due to abnormal cytology and biopsy results. Among these, 170 women were hr-HPV positive, and the remaining 58 patients were excluded. The mean age of the hr-HPV-positive subjects was 42.6 ± 10.0 years, ranging from 26 to 70 years, with the majority in the 30–39 age group. The distribution of Pap test 1 is shown in Table 1. Of them, 9 patients presented with ASCUS, 51 with LSIL, 24 with ASC-H, 83 with HSIL, 2 with AGUS, and 1 with AGC-NOS, respectively. Colposcopic examination revealed clinical suspicion of low-grade lesions in 20 cases. Additionally, 35 patients had unsatisfactory colposcopy, suggestive of potentially low-grade lesions. Colposcopic impression was confirmed in 94 patients for high-grade lesions, while 21 presented with unsatisfactory images indicative of a possible high-grade lesion.

Of the 170 patients, 101 exhibited methylation of at least one gene, while the remaining patients had negative methylation results. No cases of invalid DNA methylation were observed in our cohort. In the follow-up cytology (Pap test 2), 16 patients were negative for intraepithelial lesions or malignancy, 14 presented with ASCUS, 42 with LSIL, 14 with ASC-H, 80 with HSIL, and 4 women exhibited findings suggestive of microinvasion or invasive disease (Table 1).

Pap test 1 was indicative of a high-grade lesion in 69% (20/29) of cases with a histological diagnosis of CIN2 and in 70% (73/105) of cases with a gross diagnosis of CIN3. On the other hand, Pap test 2 indicated high-grade lesions in 65% (22/34) of CIN2 cases and 76.5% (70/92) of CIN3 cases, respectively. Regarding the histological diagnosis of the LLETZ specimen, the methylation test was positive at least in one gene in 8 of 35 cases with CIN1 (22.8%), 22 out of 34 cases with CIN2 (65%), 65 out of 92 cases with CIN3 (70.6%), and 6 out of 6 cancer cases (100%). The detailed values are presented in Table 1.

The overall diagnostic performance of Pap test 1, colposcopy, Pap test 2, DNA methylation, and the combined approach of Pap test 2 + DNA methylation based on the overall histological diagnosis or LLETZ histology is shown in Table 2. Specifically, the values of Pap test 1 and colposcopy were estimated according to the worst histopathological diagnosis. Pap test 1 demonstrated a sensitivity of 67.9%, specificity of 63.3%, positive predictive value (PPV) of 89.6%, negative predictive value (NPV) of 29.7%, and accuracy of 67.1%. On the other hand, based on the LLETZ histology, Pap test 2 showed improved sensitivity at 73.5%, with excellent specificity (97.4%), PPV (99%), and accuracy of 78.8%, though its NPV remained relatively low at 51.4%. The DNA methylation alone, also based on the LLETZ diagnosis, had a sensitivity of 69.7%, specificity of 79%, PPV of 92%, NPV of 42.9%, and accuracy of 71.8%. The combined approach of Pap test 2 and DNA methylation achieved the highest sensitivity (94.7%), specificity (76.3%), PPV (93.2%), NPV (80.6%), and overall accuracy (90.6%), indicating that the combined method is the most effective diagnostic strategy.

Table 3 illustrates a comparative analysis of the sensitivity and specificity of triage tests for detecting CIN2+ lesions, stratified by HPV16/18 and other hr-HPV types. When comparing DNA methylation with Pap test 2, there was no statistically significant difference in sensitivity across all categories. However, the specificity for detecting overall CIN2+ lesions was significantly higher for Pap test 2 (*p* = 0.039) than for DNA methylation. In contrast, the combination of DNA methylation and Pap test 2 outperformed Pap test 2 alone, showing a markedly higher sensitivity across all categories, reaching 94.5% for HPV16/18 (*p* < 0.001) and 95% for other hr-HPV CIN2+ lesions (*p* < 0.001), respectively. Specificity remained high for the combination approach (76.3%), even though lower than for Pap test 2 alone, but still statistically significant. Finally, the comparison between the combination of DNA methylation plus Pap test 2 versus DNA methylation alone further highlights the superior performance of the combined approach, with significantly higher sensitivity across all categories (*p* < 0.001) and marginal differences in specificity, particularly for other hr-HPV types (*p* = 0.0002).

**Figure 2:**
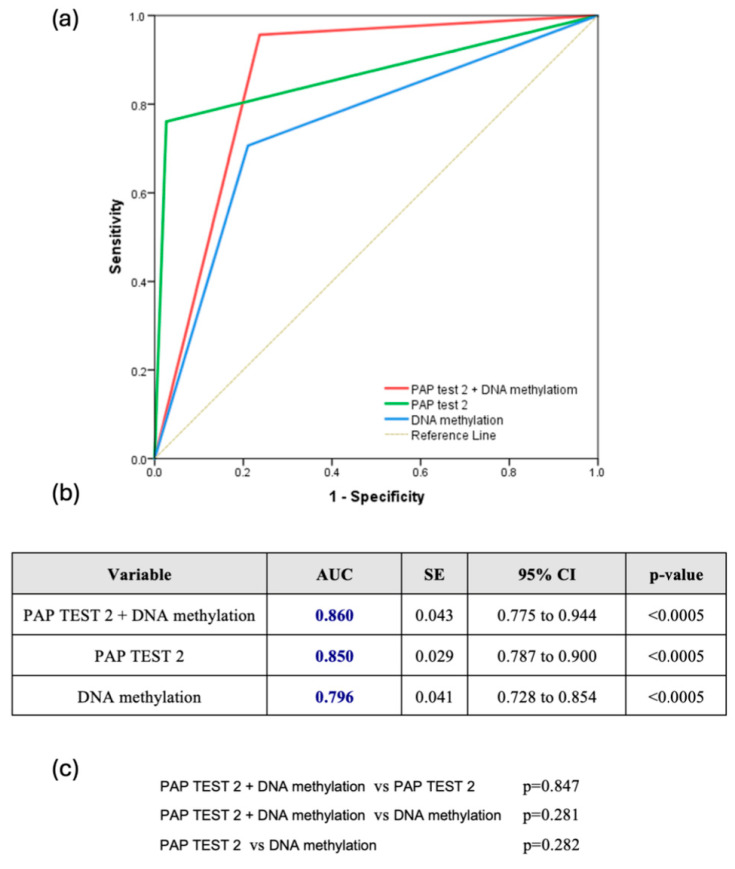
(**a**) Receiver Operating Characteristic (ROC) Curve for all hrHPV Types (ROC curve comparing the diagnostic performance of Pap test 2, DNA methylation, and their combination for detecting CIN2+ lesions across all hrHPV-positive women); (**b**) Table summarizing the area under the curve (AUC), standard error (SE), 95% confidence interval (CI), and statistical significance (*p*-value) of each test across all hrHPV-positive women; (**c**) Pairwise comparisons of AUCs between the three diagnostic strategies across all hrHPV-positive women.

**Figure 3:**
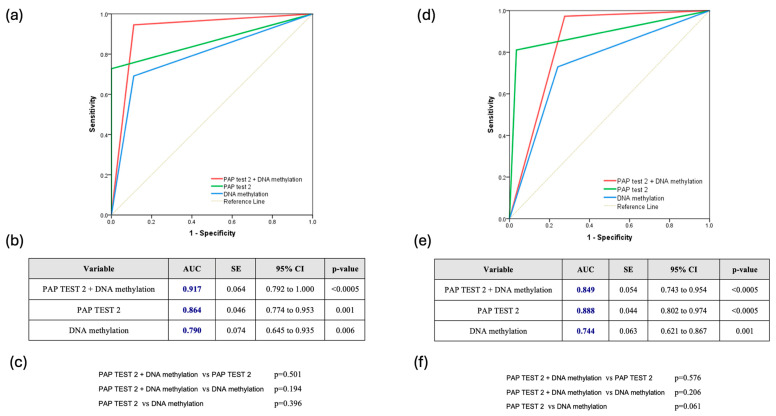
(**a**) Receiver Operating Characteristic (ROC) Curve for HPV16/18-Positive Cases (ROC curve showing the diagnostic performance of Pap test 2, DNA methylation, and their combination in hrHPV-positive women infected with HPV16/18 types); (**b**) Table summarizing the area under the curve (AUC), standard error (SE), 95% confidence interval (CI), and statistical significance (*p*-value) of each test across all hrHPV-positive women infected with HPV16/18 types; (**c**) Pairwise comparisons of AUCs between the three diagnostic strategies across all hrHPV-positive women infected with HPV16/18 types; (**d**) Receiver Operating Characteristic (ROC) Curve for Other hrHPV Types (ROC curve comparing the performance of Pap test 2, DNA methylation, and their combination in hrHPV-positive women infected with non-16/18 hrHPV types); (**e**) Table summarizing the area under the curve (AUC), standard error (SE), 95% confidence interval (CI), and statistical significance (*p*-value) of each test across all hrHPV-positive women infected with non-16/18 hrHPV types; (**f**) Pairwise comparisons of AUCs between the three diagnostic strategies across all hrHPV-positive women infected with non-16/18 hrHPV types.

The combined use of DNA methylation plus PAP test 2 consistently demonstrated superior predictive accuracy for CIN2+ lesions. Across all HPV types, the combination achieved an AUC of 0.860 (95% CI: 0.775–0.944), slightly outperforming PAP test 2 (AUC: 0.850, 95% CI: 0.787–0.900) and DNA methylation (AUC: 0.796, 95% CI: 0.728–0.854), though the differences were not statistically significant (Figure 2a–c). For HPV16/18 infections, the combined approach was also particularly effective, reaching an AUC of 0.917 (95% CI: 0.792–1.000), compared to 0.864 for PAP test 2 and 0.790 for DNA methylation, without significant differences (*p* = 0.501 and *p* = 0.194, respectively) (Figure 3a–c). Finally, for other high HPV types, the combined approach demonstrated strong predictive performance with an AUC of 0.849 (95% CI: 0.743–0.954), closely rivaling PAP test 2, which achieved a slightly higher AUC of 0.888 (95% CI: 0.802–0.974). DNA methylation, while effective, showed comparatively lower accuracy with an AUC of 0.744 (95% CI: 0.621–0.867). However, once again, the comparisons among the methods did not reveal any statistically significant difference favoring one over the others (Figure 3d–f).

## 4. Discussion

### 4.1. Principal Findings

The implementation of hr-HPV testing as a primary screening method in national screening programs improves the risk stratification by increasing the sensitivity for the detection of CIN2+ compared to cytology, although there are considerable limitations in its specificity. Additional biomarkers were tested alone or in combination to overcome this limitation. DNA methylation assays seem to contribute, as a triage method, to CIN2+ detection in the hr-HPV positive population. Our results suggest that the combination of an additional cervical cytology (Pap test 2) with a DNA methylation test can be a reliable triage method in hr-HPV positive women with high sensitivity (94.7%) and specificity (76.3%), and considerably better than cytology alone.

### 4.2. Comparison with Existing Literature

The diagnostic performance of DNA methylation markers in our study reflects moderate diagnostic accuracy compared to existing literature. Vink et al. reported AUC values consistently exceeding 0.85 when methylation markers such as FAM19A4 and miR124-2 were utilized, with sensitivities often above 80% in high-risk populations [16]. Similarly, Gu et al. demonstrated a sensitivity of 89.1% and an AUC of 0.86 using the S5 methylation classifier, reinforcing the robust diagnostic potential of these assays [17]. While specificity in our study aligns with many previous findings, such as Zhang et al., who observed similar specificity rates in hr-HPV-positive cohorts, the slightly lower sensitivity emphasizes the variability of performance metrics depending on study design and population characteristics, given the use of LSIL patients as the control group in our study, which increases the overlap of methylation profiles between cases and controls [18]. Overall, methylation markers continue to exhibit consistent diagnostic reliability across diverse settings, with outcomes influenced by methodological and population-specific factors.

The differential performance of methylation markers between HPV16/18-positive individuals and those with other hr-HPV types is a noteworthy finding in our study. For HPV16/18-positive women, the sensitivity reached 71.2% and specificity 88.9%, in agreement with Gu et al., who reported sensitivities exceeding 90% for these subtypes in controlled cohorts [17]. Similarly, Verhoef et al. reported an AUC of 0.86 for methylation alone in detecting CIN3+ lesions in HPV16/18-positive cases [19]. In the current study, methylation alone achieved a comparable AUC of 0.796 for CIN2+ lesions across all hrHPV-positive women and an even higher AUC of 0.917 when stratified to HPV16/18-positive cases. The superior performance for HPV16/18 may be attributed to the higher oncogenic potential and more consistent epigenetic changes associated with these subtypes, as also noted by Vink et al., who highlighted the enhanced accuracy of methylation assays in this subgroup [20]. In this context, for other hr-HPV types, our study observed reduced sensitivity (67.8%) and specificity (75.9%), in accordance with reports suggesting that these subtypes exhibit greater variability in methylation profiles. This variability underscores the potential need for tailored methylation panels to optimize detection across HPV genotypes. Despite these differences, our findings confirm the broad applicability of methylation markers, while identifying areas where further optimization is needed to enhance their diagnostic utility.

Age-dependent variability in the performance of methylation markers has been well-documented in the literature. Leeman et al. reported that methylation assays demonstrate reduced sensitivity in younger women, likely due to lower levels of methylation associated with early-stage lesions in this age group [21]. Similarly, Zhang et al. emphasized that the performance of methylation markers can differ significantly across age groups, emphasizing the importance of age-stratified analyses in optimizing diagnostic accuracy [18]. In the current study, age stratification was not conducted, which could partially explain the variability in sensitivity and specificity observed. Future studies should explore the diagnostic performance of methylation markers across different age cohorts to better understand their potential limitations and refine their clinical applicability. This kind of stratification could help identify whether methylation can be used alone in special subgroups or may require complementary approaches for optimal results.

The combination of methylation markers with Pap test yielded significant improvements in diagnostic performance, achieving an overall sensitivity of 94.7% and specificity of 76.3% with an AUC of 0.860. This combined approach enhances the strengths of each method, as also demonstrated in the post hoc analysis of the POBASCAM trial, where the addition of methylation to cytology reduced unnecessary colposcopy referrals without compromising sensitivity [22]. Similarly, Zhang et al. emphasized the value of integrating methylation assays with traditional methods, noting that this combination provides a more robust strategy for triaging hr-HPV-positive women [18]. Our findings further support the utility of integrated testing strategies in clinical practice; however, as highlighted in several studies, the variability in methodologies, cutoff thresholds, and populations studied necessitates further standardization to ensure consistency and reproducibility across diverse healthcare settings [23].

### 4.3. Clinical Implications

This study highlights the significant potential of DNA methylation as a tool for cervical cancer screening and triage. Unlike cytological assessments such as the Pap test or the dual-stain method (p16/Ki-67 immunostaining), the methylation test, a molecular method, eliminates subjectivity arising from cytologist expertise and sample collection techniques, ensuring consistent and reproducible results. This is also evident in our findings, where Pap test 2, received by a specialized gynecologist, achieved higher sensitivity (73.5% vs. 67.9%) and specificity (97.4% vs. 63.3%) than Pap test 1. Moreover, methylation offers a unique prognostic advantage, as it can identify lesions with potential progression to high-grade lesions or malignancy. This capability surpasses methods like the p16/Ki-67 dual-stain cytology, which are limited to detecting existing abnormalities without assessing future risk. This ability to predict disease progression is particularly valuable in stratifying patients for appropriate follow-up and minimization of overtreatment [24].

DNA methylation does not require high cellularity, which presents distinct clinical advantages. This allows for the use of self-sampling methods, making screening more accessible and patient-friendly. This has been further supported by De Strooper et al., who demonstrated that FAM19A4 and hsa-miR124-2 methylation testing maintains high performance in both lavage- and brush-based self-collected samples from hrHPV-positive women, reinforcing the applicability of methylation analysis in broader screening contexts [25]. Moreover, the same sample can be used to perform other tests, such as HPV testing or cytological tests, alongside methylation analysis, optimizing resources, simplifying laboratory workflows, and significantly improving the diagnostic accuracy of triage for high-grade lesions and cervical cancer. However, while the combination of HPV genotyping, cytology, and methylation analysis may optimize diagnostic performance, it is anticipated to be associated with increased costs compared to existing, simpler triage protocols. Therefore, its overall cost-effectiveness in large-scale screening programs remains to be determined. Future studies incorporating formal cost-benefit analyses will be essential to assess whether the improvement in diagnostic accuracy justifies the additional resource utilization in routine clinical practice.

In our study, the combination of DNA methylation with the Pap test enhanced diagnostic accuracy in triaging hrHPV-positive patients. Specifically, the combined approach achieved a sensitivity of 94.7% and specificity of 76.3% for CIN2+ lesions, demonstrating the complementary strengths of combined testing. The integration of these methods improves specificity, thus refining triage strategies and minimizing unnecessary colposcopies. In this context, the combined use of Pap test and DNA methylation could be proposed as an approach for patients with hrHPV infections from non-16/18 subtypes. Particularly, in our study this method demonstrated a sensitivity of 95%, specificity of 72.4%, positive predictive value (PPV) of 87.5%, negative predictive value (NPV) of 87.5%, and an area under the curve (AUC) of 0.849, which are comparable to the corresponding results reported in the literature for the p16/Ki-67 dual-stain cytology [26,27,28].

### 4.4. Strengths and Limitations

The present study has several notable strengths. First, its prospective design ensures high-quality data collection and minimizes biases, enhancing the validity of the findings. Additionally, it is among the few studies in the international literature that investigate the combination of DNA methylation with cytology, offering novel insights into the complementary strengths of these methods for the triage of precancerous lesions of the cervix. Furthermore, the use of the QIAsure^®^ test, a well-validated and reproducible methylation assay with robust inter- and intra-laboratory reliability, adds significant credibility to the results. Finally, by focusing on a population of hr-HPV-positive women, the study aligns closely with real-world clinical scenarios, making the findings particularly applicable and reproducible for the development of effective triage strategies for high-grade intraepithelial lesions or cervical cancer.

Despite its strengths, this study has certain limitations that warrant consideration. As a single-center study with a relatively small sample size, the generalizability of the findings to broader populations may be limited. Additionally, the small number of cases with cervical cancer and the limited representation of isolated CIN2 and CIN3 lesions precluded stratification based on specific types of high-grade abnormalities. Consequently, the results were analyzed collectively for a single group of CIN2+ lesions, as the relatively small sample size did not allow for separate, adequately powered analyses of CIN2 and CIN3 cases. This necessary grouping may have overlooked important diagnostic differences between these two histological stages, potentially limiting the granularity of the findings. Moreover, the choice of LSIL patients as the control group, rather than healthy individuals with no abnormalities, may have influenced the diagnostic performance metrics. Specifically, this selection likely reduced specificity and NPV while inflating PPV, as LSIL lesions share some biological characteristics with higher-grade abnormalities, thereby narrowing the diagnostic margin. These factors should be considered when interpreting the sensitivity, specificity, and predictive values reported in the study. An additional limitation stems from the exclusion of women who were HR-HPV negative on post-biopsy ThinPrep^®^ samples despite having biopsy-proven CIN2+ lesions. This necessary methodological choice, in order to reflect the intended screening scenario focused on HR-HPV-positive populations, may have introduced selection bias by enriching the study population with cases showing optimal diagnostic agreement. Moreover, the performance of colposcopy-guided biopsies prior to sample collection could have reduced the detectable viral load, potentially influencing the subsequent results of both HPV testing and methylation analysis. Finally, the relatively small sample size limited our ability to perform stratified analyses based on age groups. As methylation marker performance may vary with age, particularly among younger women, this limitation should be considered when interpreting our findings. Addressing these limitations in future research could further enhance the robustness and applicability of the findings.

## 5. Conclusions

DNA methylation, especially when combined with cytology, demonstrates strong potential as a triage tool for high-grade cervical lesions in hrHPV-positive women. Its integration into screening protocols could refine diagnostic accuracy and optimize patient management. This approach addresses key limitations of current methods and offers a reproducible, patient-centered solution for cervical cancer prevention. However, further research is needed to validate these findings in larger, more diverse populations.

## Figures and Tables

**Table 1:** Screening Test Results in Correlation with Histological Diagnosis.

		Overall Histological Diagnosis (*n*, %)				
Cytology 1 (Pap Test 1) (*n*, %)		Negative	CIN 1	CIN 2	CIN 3	CA
		*n* = 0	*n* = 30	*n* = 29	*n* = 105	*n* = 6
ASCUS	9 (**5.3%**)	−	18	9	32	1
LSIL	51 (**30%**)					
ASC-H	24 (**14.1%**)	−	12	20	73	5
HSIL	83 (**48.8%**)					
AGUS	2 (**1.2%**)					
AGC-NOS	1 (**0.6%**)					
**Colposcopy (*n*, %)**						
LSIL	20 (**11.8%**)	−	6	7	7	0
LSIL UN	35 (**20.6%**)	−	18	4	13	0
HSIL	94 (**55.3%**)	−	6	16	71	1
HSIL UN	21 (**12.3%**)	−	0	2	14	5
		**LLETZ Histological Diagnosis (*n*, %)**				
**Cytology 2 (Pap test 2) (*n*, %)**		**Negative**	**CIN 1**	**CIN 2**	**CIN 3**	**CA**
		*n* = **3**	*n* = **35**	*n* = **34**	*n* = **92**	*n* = **6**
NILM	16 (**9.4%**)	3	35	12	22	0
ASCUS	14 (**8.2%**)					
LSIL	42 (**24.7%**)					
ASC-H	14 (**8.2%**)	0	0	22	70	6
HSIL	80 (**47.1%**)					
Suspicion of Invasion	3 (**1.8%**)					
Carcinoma	1 (**0.6%**)					
**DNA Methylation (*n*, %)**						
QIAsure Methylation test positive	101 (**59.4%**)	0	8	22	65	6
QIAsure Methylation test negative	69 (**40.6%**)	3	27	12	27	0

Abbreviations: ASCUS: Atypical squamous cells of undetermined significance; LSIL: Low-grade squamous intraepithelial lesions; ASC-H: Atypical squamous cells, cannot exclude high-grade squamous intraepithelial lesion; HSIL: High-grade squamous intraepithelial lesions; AGUS: atypical glandular cells of undetermined significance; AGC-NOS: atypical glandular cells, not otherwise specified; UN: Unsatisfactory; CIN: Cervical Intraepithelial Neoplasia; CA: Carcinoma.

**Table 2:** Performance Metrics of Triage Tests for CIN2+ Stratified by HPV Test Results.

	Pap Test 1 *	Colposcopy *	Pap Test 2 **	DNA Methylation **	Pap Test 2 + DNA Methylation **
**Sensitivity**	**Overall**:	**Overall**:	**Overall**:	**Overall**:	**Overall**:
	67.9% (60–76%)	75.7% (68–83%)	73.5% (65–81%)	69.7% (61–77%)	94.7% (89–98%)
	**HPV 16/18**:	**HPV 16/18**:	**HPV 16/18**:	**HPV 16/18**:	**HPV 16/18**:
	63.0% (51–74%)	79.5% (68–88%)	71.2% (59–81%)	71.2% (59–81%)	94.5% (85–98%)
	**Other hr-HPV**:	**Other hr-HPV**:	**Other hr-HPV**:	**Other hr-HPV**:	**Other hr-HPV**:
	73.1% (61–83%)	71.6% (59–82%)	76.3% (63–86%)	67.8% (54–79%)	95% (85–99%)
**Specificity**	**Overall**:	**Overall**:	**Overall**:	**Overall**:	**Overall**:
	63.3% (44–80%)	83.3% (65–94%)	97.4% (86–100%)	79% (63–91%)	76.3% (59–88%)
	**HPV 16/18**:	**HPV 16/18**:	**HPV 16/18**:	**HPV 16/18**:	**HPV 16/18**:
	77.8% (40–97%)	88.9% (52–100%)	100% (66–100%)	88.9% (52–100%)	88.9% (51–99%)
	**Other hr-HPV**:	**Other hr-HPV**:	**Other hr-HPV**:	**Other hr-HPV**:	**Other hr-HPV**:
	57.1% (34–78%)	81% (58–95%)	96.6% (82–100%)	75.9% (56–89%)	72.4% (53–87%)
**Positive Predictive Value**	**Overall**:	**Overall**:	**Overall**:	**Overall**:	**Overall**:
	89.6% (84–93%)	95.5% (91–98%)	99% (93–100%)	92% (86–96%)	93.2% (87–97%)
	**HPV 16/18**:	**HPV 16/18**:	**HPV 16/18**:	**HPV 16/18**:	**HPV 16/18**:
	95.83% (87–99%)	98.3% (90–100%)	100%	98.1% (89–100%)	98.6% (91–100%)
	**Other hr-HPV**:	**Other hr-HPV**:	**Other hr-HPV**:	**Other hr-HPV**:	**Other hr-HPV**:
	84.5% (77–90%)	92.3% (83–97%)	97.8% (87–100%)	85.1% (75–92%)	87.5% (76–94%)
**Negative Predictive Value**	**Overall**:	**Overall**:	**Overall**:	**Overall**:	**Overall**:
	29.7% (23–37.8%)	42.4% (35–51%)	51.4% (44–58%)	42.9% (36–51%)	80.6% (63–91%)
	**HPV 16/18**:	**HPV 16/18**:	**HPV 16/18**:	**HPV 16/18**:	**HPV 16/18**:
	20.6% (14–29%)	34.8% (24–47%)	30% (23–38%)	27.6% (20–37%)	66.7% (35–89%)
	**Other hr-HPV**:	**Other hr-HPV**:	**Other hr-HPV**:	**Other hr-HPV**:	**Other hr-HPV**:
	40.0% (28–53%)	47.2% (37–58%)	66.7% (56–76%)	53.7% (43–64%)	87.5% (66–97%)
**Accuracy**	**Overall**:	**Overall**:	**Overall**:	**Overall**:	**Overall**:
	67.1% (59–74%)	77.1% (70–83%)	78.8% (71–85%)	71.8% (64–78%)	90.6% (85–95%)
	**HPV 16/18**:	**HPV 16/18**:	**HPV 16/18**:	**HPV 16/18**:	**HPV 16/18**:
	64.4% (53–75%)	80.5% (70–88%)	74.4% (64–83%)	73.2% (62–82%)	93.9% (86–98%)
	**Other hr-HPV**:	**Other hr-HPV**:	**Other hr-HPV**:	**Other hr-HPV**:	**Other hr-HPV**:
	69.3% (59–78%)	73.9% (63–83%)	83% (73–90%)	70.5% (60–79%)	87.5% (79–94%)

Abbreviations: *: The variables are estimated according to the overall worst diagnosis, **: The variables are estimated according to the cone biopsy.

**Table 3:** Comparative Analysis of Sensitivity and Specificity of Triage Tests for CIN2+ Lesions.

Comparison	Sensitivity			Specificity		
**DNA Methylation vs. Pap Test 2**	**Overall**	**HPV16/18**	**Other hr-HPV**	**Overall**	**HPV16/18**	**Other hr-HPV**
DNA Methylation	69.7% (61–77%)	71.2% (59–81%)	67.8% (54–79%)	79% (63–91%)	88.9% (52–100%)	75.9% (56–89%)
Pap Test 2	73.5% (65–81%)	71.2% (59–81%)	76.3% (63–86%)	97.4% (86–100%)	100% (66–100%)	96.6% (82–100%)
*p*-values	*0.609*	*1.000*	*0.441*	***0.039***	*1.000*	*0.07*
**DNA Methylation + Pap test 2 vs. ** **Pap Test 2**	**Overall**	**HPV16/18**	**Other hr-HPV**	**Overall**	**HPV16/18**	**Other hr-HPV**
DNA Methylation + Pap test 2	94.7% (89–98%)	94.5% (85–98%)	95% (85–99%)	76.3% (59–88%)	88.9% (51–99%)	72.4% (53–87%)
Pap Test 2	73.5% (65–81%)	71.2% (59–81%)	76.3% (63–86%)	97.4% (86–100%)	100% (66–100%)	96.6% (82–100%)
*p*-values	***0.0005***	***0.0001***	***0.003***	***0.023***	*1.000*	***0.023***
**DNA Methylation + Pap test 2 vs. DNA Methylation**	**Overall**	**HPV16/18**	**Other hr-HPV**	**Overall**	**HPV16/18**	**Other hr-HPV**
DNA Methylation + Pap test 2	94.7% (89–98%)	94.5% (85–98%)	95% (85–99%)	76.3% (59–88%)	88.9% (51–99%)	72.4% (53–87%)
DNA Methylation	69.7% (61–77%)	71.2% (59–81%)	67.8% (54–79%)	79% (63–91%)	88.9% (52–100%)	75.9% (56–89%)
*p*-values	***0.0001***	***0.0001***	***0.0002***	*0.480*	*1.000*	*1.000*

## Data Availability

The original contributions presented in this study are included in the article. Further inquiries can be directed to the corresponding author(s).

## Abbreviations

The following abbreviations are used in this manuscript:

AGC	Atypical Glandular Cells
AGC-NOS	Atypical Glandular Cells-Not Otherwise Specified
AGUS	Atypical Glandular of Undetermined Significance
ASC-H	Atypical Squamous Cells-cannot exclude HSIL
ASCUS	Atypical Squamous of Undetermined Significance
AUC	Arean Under the Curve
CIN	Cervical Intraepithelial Neoplasia
HPV	Human Papillomavirus
hr-HPV	High risk Human Papillomavirus
HSIL	High-grade Squamous Intraepithelial Lesion
LEEP	Loop Electrosurgical Excision Procedure
LLETZ	Large Loop Excision of the Transformation Zone
LSIL	Low-grade Squamous Intraepithelial Lesion
NPV	Negative Predictive Value
PPV	Positive Predictive Value
ROC	Receiver Operating Characteristic
Se	Sensitivity
Sp	Specificity
